# A Novel Combined Audiovisual-Semantic Digital Tool for Early-Stage Cognitive Decline Detection: Development and Validation Study

**DOI:** 10.2196/91165

**Published:** 2026-05-05

**Authors:** Qiwei Wu, Binyu Zhao, Zihao Zheng, Betsy Seah, Xueping Zhang, Jing Shao

**Affiliations:** 1 Department of Nursing, The Fourth Affiliated Hospital of School of Medicine, and International School of Medicine, International Institutes of Medicine, Zhejiang University, Hushan Road 200, Yiwu, Zhejiang Province, China; 2 Institute of Nursing Research, Zhejiang University School of Medicine, Hangzhou, Zhejiang, China; 3 Alice Lee Centre for Nursing Studies, Yong Loo Lin School of Medicine, National University of Singapore, Singapore; 4 Department of Geriatric Psychiatry, The Seventh People’s Hospital of Hangzhou, Mental Health Center of Zhejiang University School of Medicine, Hangzhou, Zhejiang, China

**Keywords:** cognitive decline, working memory, audiovisual integration, semantic categorization, go/no-go task, community screening

## Abstract

**Background:**

Dementia poses a significant public health challenge. Early detection is crucial for timely intervention but remains difficult in community settings, as many existing cognitive screening tools are either insufficiently sensitive to subtle decline or too burdensome for widespread use.

**Objective:**

This study aimed to develop and validate a novel combined audiovisual-semantic digital tool for rapid and acceptable community-based screening of cognitive decline in older adults.

**Methods:**

A total of 156 older adults completed 6 progressively complex task variants shifting from unimodal to multimodal stimuli and from basic to superordinate semantic categorization. Performance was measured using reaction time, accuracy, false alarms, and inverse efficiency. Diagnostic utility was assessed via logistic regression and receiver operating characteristic curve analysis, whereas qualitative interviews evaluated acceptability.

**Results:**

Task outcomes showed significant declines across the cognitive continuum from no cognitive impairment to mild cognitive impairment to dementia (*P*<.001). The integrated audiovisual-semantic condition at the basic categorization level reliably differentiated cognitive groups and achieved higher area under the curve values for distinguishing no cognitive impairment from cognitive impairment and mild cognitive impairment from dementia compared to basic-level tasks. Incorporating semantic processing enhanced diagnostic discrimination. Participant feedback was highly positive, with 48.7% (76/156) describing the tasks as “fun/interesting.”

**Conclusions:**

The audiovisual-semantic integrated digital tool is a valid, well-accepted, and time-efficient instrument for cognitive screening in older adults. Its design, which increases cognitive load through multimodal and semantic integration, improves sensitivity to early decline, supporting its potential for practical community application.

## Introduction

Dementia is one of the major causes of disability and dependency among older people‚ posing a significant public health challenge worldwide [1]. While dementia currently lacks effective treatment, it is understood to be a progressive continuum. There is cumulative evidence suggesting that early identification of and intervention for cognitive decline confer both clinical and health-economic benefits [2]. Notably, approximately 14.4% to 38% of individuals with mild cognitive impairment (MCI) who are identified through screening and receive interventions revert to normal cognitive functioning [3,4]. Consequently, screening tools with high specificity and sensitivity are critical. Current assessment methods, which primarily include physiological indicators, neuroimaging techniques, and questionnaires, are often invasive, costly, and time-consuming [5]. In contrast, some digital tools (eg, voice based or virtual reality [VR]) show substantial clinical promise by addressing these shortcomings [6,7]. However, these new tools still have limitations (eg, cybersickness in VR and accent-related transcription errors) and require further exploration [5,6,8].

Impairment of working memory is a hallmark early symptom of dementia [9]. Working memory is a limited-capacity cognitive system that provides temporary storage and manipulation of information to support a wide range of complex activities such as reasoning, comprehension, and learning [10]. Neuroimaging studies have demonstrated altered activation in working memory–related brain regions during cognitive tasks in the early disease continuum [11,12]. However, translating these findings into early diagnostic tools remains a challenge as it hinges on deconstructing working memory into its measurable component processes. The multicomponent model of working memory by Baddeley [10] offers a simple yet robust framework that makes such deconstruction possible. This proposed framework comprises 4 core components: the central executive system, phonological loop, visuospatial sketchpad, and episodic buffer [13]. External sensory input is initially handled by the phonological loop and visuospatial sketchpad. The episodic buffer then binds this information with existing knowledge from long-term memory. Finally, the central executive system acts as the control system, achieving cognitive processes by regulating attention, updating information, and inhibiting irrelevant information [13]. Previous research has reported that inhibitory control represents the most frequently impaired executive domain in MCI [14]. Indeed, the central executive system has been confirmed as an interconnected network where inhibitory control relies on attention, inhibition, and other functions that are integrated across neural networks in an interactive manner [14]. Furthermore, the association between working memory capacity and higher-order cognitive abilities is more pronounced, with these differences largely attributed to attentional capacity [15]. Therefore, researchers believe that access to working memory is governed by inhibitory attention processes [16].

The go/no-go task is widely used as a measure of inhibition, attention, and impulsivity [17]. By designing tasks that require participants to respond to specific stimuli (“go”) and withhold responses to “no-go” stimuli, which assesses the ability of executive control, extensive behavioral and neuroimaging studies have demonstrated the neurobiological foundation of the go/no-go task [17-19]. Cognitive control–related tasks consistently elicit increased frontal midline θ power, which is a key neural marker for cognitive control, response inhibition, conflict monitoring, and attention allocation [18]. The increase in frontal midline θ power during go/no-go tasks is driven by both inhibitory and noninhibitory (attention-related) processing [19]. Furthermore, rapid response inhibition activates the prefrontal cortex (PFC), particularly the middle and inferior frontal gyri, as well as the parietal and temporal lobes and their functional networks [20]. Abnormalities in these regions are considered predictive of cognitive status [20]. Significantly, the PFC is particularly vulnerable to aging [21]. Some researchers have even proposed the frontal or executive decline hypothesis, suggesting that age-related cognitive decline may stem from damage to the prefrontal lobe and executive functions [22]. Therefore, the go/no-go task is an experimental paradigm that effectively reflects executive control and has been reported to be a potential practical early screening tool for cognitive decline [23,24].

According to the model by Baddeley [10], processing stimuli involves a multisensory operation that necessitates cross-modal integration within the episodic buffer. However, go/no-go tasks predominantly use visual stimuli [25]. Given that sensory function decline is a natural consequence of aging, relying on a single sensory modality may introduce measurement bias. Previous research has indicated that multisensory integration accelerates reaction time (RT) in older adults, potentially serving as a compensatory mechanism to enhance brain information processing efficiency [26]. Beyond facilitation effects, multisensory processing exhibits illusory effects such as the McGurk effect and the double-flash illusion [27]. These illusory effects are mediated by anatomical and functional connections between different brain regions, particularly the cross-modal associations involving neural resources in the PFC [28]. Cross-modal information is represented within the capacity-constrained episodic buffer, where more complex inputs entail the binding of a greater number of information sources, thereby elevating processing load [29]. Moreover, multisensory integration can be affected by top-down factors, also termed endogenous attention [30]. Endogenous attention requires purposeful and effort-intensive cognitive processing, whereas multisensory integration performance may exhibit instability or illusions under high–cognitive load conditions.

Semantic memory is a key component of long-term memory. Its integration with working memory occurs in the episodic buffer, followed by complex processing in the central executive system to support motor behavior. Prior research suggests that working memory is not related to basic sensory processing (eg, color or shape discrimination) but shows a reliable association with semantic tasks [31]. A meta-analysis revealed that semantic deficits constitute a crucial and early feature of MCI and should be incorporated into routine clinical assessments [32]. Furthermore, the complexity of conceptual semantic representations systematically influences inhibitory processing [33]. However, most published go/no-go studies measure perceptual discrimination of stimuli such as shapes or numbers, with little attention paid to changes in abstract concepts [34]. This neglect may result in insufficient sensitivity of go/no-go tasks in capturing early and subtle cognitive decline.

To sum up, based on the theoretical framework by Baddeley [10] and existing empirical evidence, we hypothesize that combining visual-auditory fusion and semantic categorization with the go/no-go task can heighten demand on central executive resources, which might lead to a better identification of cognitive decline. The aim of this study was to develop an integrated audiovisual-semantic go/no-go task and evaluate its performance in discriminating cognitive decline, as well as the task’s acceptability among older adults.

## Methods

### Study Design and Participants

This cross-sectional pilot study was conducted to preliminarily investigate and develop a digital tool integrating audiovisual-semantic stimuli for the detection of cognitive decline. From April 2025 to August 2025, a total of 156 participants aged 60 years and above were recruited from 7 communities in Hangzhou, China. Exclusion criteria were (1) individuals who had significant verbal and hearing impairments and were unable to see or hear the stimuli despite adjusting the volume or image size (by controlling the resolution in the E-Prime software [Psychology Software Tools]) and (2) those with major depression and other psychiatric disorders.

### Data Collection

#### Overview

Data collection was conducted during a single session. All participants or their legally acceptable representatives signed a written consent form agreeing to take part in this study. After that, all participants underwent face-to-face interviews conducted by a trained staff member to collect the demographic information (eg, age and gender). We administered the Montreal Cognitive Assessment (MoCA) and the go/no-go tasks in an interleaved and alternating manner using a number-calling strategy, with at least a 30-minute interval between the 2 assessments. The MoCA assessment was conducted by trained research personnel.

#### Cognitive Assessment

The MoCA is a well-validated, multidomain, paper-and-pencil test that assesses a range of cognitive functions, including visuospatial and executive functions, naming, memory, attention, abstraction, and orientation [35]. We defined participants’ cognitive status using the following MoCA cutoff scores (adjusted education; the MoCA should be scored based on educational level to reduce bias. According to the standard MoCA scoring rules, one point is added to the total score for participants with less than 6 years of education): a score of 23 or higher was categorized as no cognitive impairment (NCI), a score ranging from 14 to 22 was categorized as MCI, and a score of less than 14 was categorized as dementia [5].

#### The Modified Go/No-Go Task

**Figure 1.**
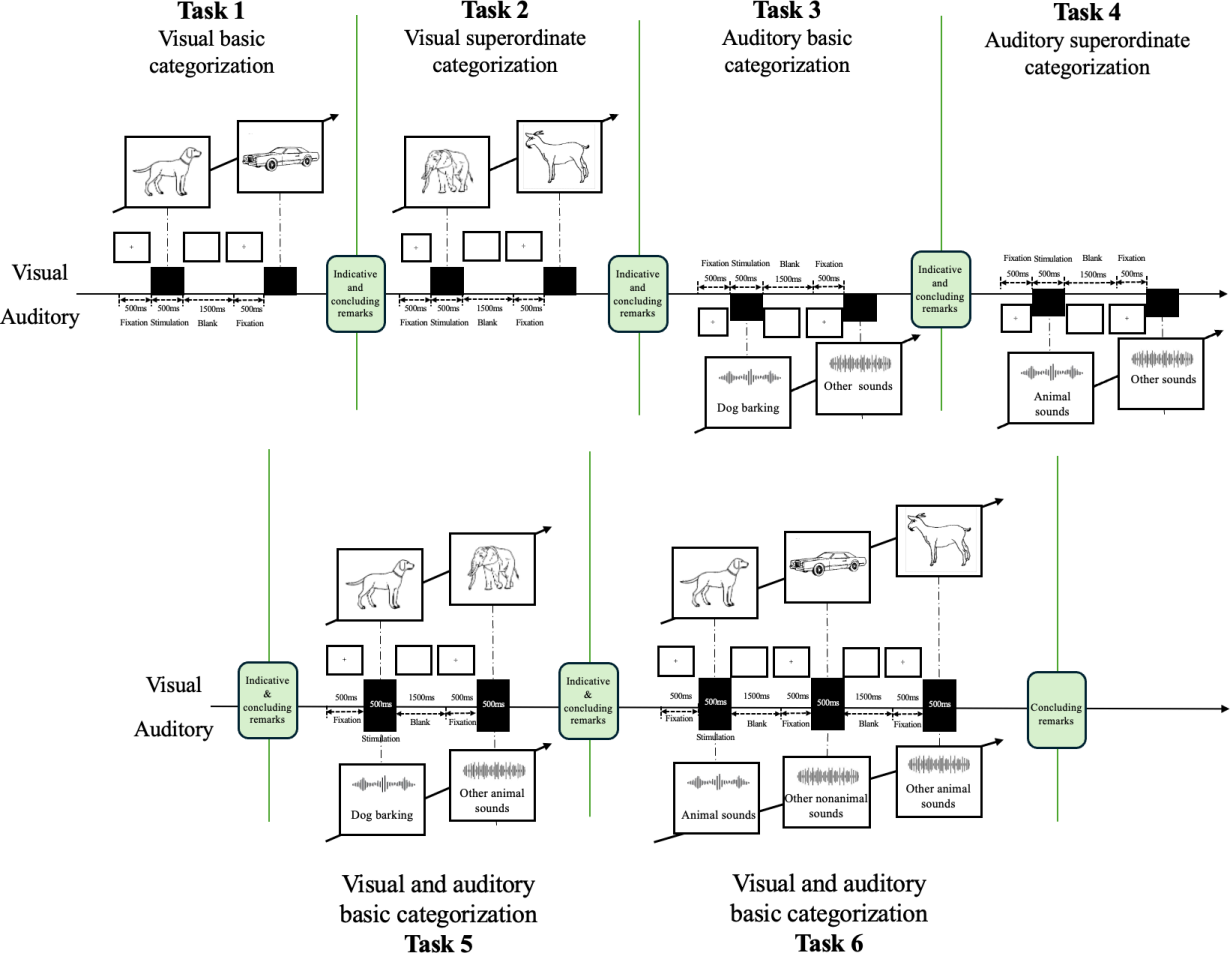
Go/no-go tasks.

The task design was built on previous research and finalized through consensus among all authors [24]. This study comprised 6 go/no-go tasks (Figure 1), including visual, auditory, and audiovisual integration tasks. Every task consisted of 5 runs of 16 trials each (80 total trials). The total completion time for the 6 tasks was approximately 18 minutes (170 seconds per task). Participants were allowed a 1-minute break after every task. The tasks were conducted in a quiet environment (≤45 dB), verified using a decibel meter (model DLX-PS02303; Delixi Electric). Participants sat at approximately 58 cm from a 14-inch laptop computer (ThinkPad T480s-i7; Lenovo). Auditory stimuli were presented through wired headphones (model HD350BT; Sennheiser Electronic SE & Co). Volume was adjusted to a level clearly audible to participants before testing commenced. Visual materials were sourced from a canonical set of line drawings [36], and auditory materials were obtained from the Aigei website. As prior research has suggested that humans are more sensitive to biological sounds compared to nonbiological sounds, the auditory stimuli were designed as animal sound recognition tasks [37]. Visual and auditory stimulus materials underwent processing to standardize the image size and sound pressure level before being inserted into E-Prime (version 2.0). The sequence of stimuli in each task was fixed. A single-key keyboard (model K1-LY-WX; Yingyu IoT) was connected to the computer and positioned in front of the participant’s dominant hand. Each stimulus was presented for 500 ms, with a 1500-ms intertrial interval during which a blank screen with a fixation cross was displayed. Each task was preceded by 12 practice trials at the same pace as the formal study trials (go/no-go ratio of 3:1) to ensure participant comprehension of task requirements. The go/no-go metrics evaluated were as follows:

- RT: mean response time for correct “go” trials
- “Go” trial accuracy: percentage of correct responses on “go” trials
- False alarm rate: percentage of incorrect responses on “no-go” trials
- Inverse efficiency score: RT divided by the overall proportion of correct responses

A lower score reflects better inhibitory control by integrating speed and accuracy. All go/no-go metrics were standardized into *Z*-scores before performing regression analysis.

#### Informal Interview

Following completion of the MoCA and modified go/no-go tasks, we conducted informal interviews lasting approximately 3 to 5 minutes with participants. Semistructured questions included the following: “How did you feel about the overall testing process of Go/No-go?” “How was your experience with the Go/No-go task compared with the MoCA questionnaire?” “How do you feel about implementing this Go/No-go task for cognitive decline screening in a community setting?” Interviewers recorded key content words related to feelings, with each unique word recorded only once per participant.

### Data Analysis

Statistical analyses were performed using SPSS (version 31; IBM Corp). As the data in this study did not pass normality tests, medians and IQRs were used for description. Kruskal-Wallis tests were used for demographic characteristics and go/no-go task–related metric analyses. Logistic regression analyses were conducted while controlling for age and educational level (*P*<.05 in both cases). We used backward likelihood ratio logistic regression analysis, with a *P* value of .05 for entry and .10 for removal. Backward likelihood ratio is considered more robust based on overall model fit changes [38]. The features extracted from the task were entered into a logistic regression model, where the diagnostic group (NCI vs cognitive impairment, MCI, or dementia and MCI vs dementia) was considered as the dependent variable and the task-extracted features were used as the independent variables.

To examine go/no-go diagnostic validity, receiver operating characteristic (ROC) curves were used to establish area under the curve (AUC) values for the following groups: NCI vs cognitive impairment (cognitive impairment including MCI and dementia), NCI vs MCI, NCI vs dementia, and MCI vs dementia. The optimal Youden index was used to calculate discrimination metrics, including sensitivity and specificity. AUC 95% CIs were calculated using bootstrap methods. To compare the discriminative validity of different tasks, additional ROC analyses were performed using the DeLong method. To analyze the qualitative data, the R package *wordcloud2* (R Foundation for Statistical Computing) was used to generate word clouds.

### Ethical Considerations

All procedures were approved by the Ethics Committee for Human Studies of the Fourth Affiliated Hospital of Zhejiang University School of Medicine (2025-0326-1-F1). Written informed consent was obtained from all participants prior to measurements, in accordance with the Declaration of Helsinki. No identifying details are included in this paper or the supplementary materials. Participants did not receive compensation. Access to raw data is restricted to research team members.

## Results

### Characteristics of the Participants

A total of 156 participants took part in this study, including those with NCI (n=75, 48.1%), MCI (n=66, 42.3%), and dementia (n=15, 9.6%). Baseline characteristics (Table 1) showed significant differences among the 3 groups in age, educational level, and MoCA scores (*P*<.001). No statistically significant differences were observed in gender or smoking or alcohol consumption habits across the 3 groups.

**Table 1.** Participant characteristics (N=156).

	NCI^a^ (n=75)	MCI^b^ (n=66)	Dementia (n=15)	Total	*P* value
Age (y), median (IQR)	70 (65-77)	76 (71-80)	82 (73-87)	74 (68-79)	<.001^c^
Sex (female), n (%)	23 (30.7)	13 (19.7)	1 (6.7)	37 (23.7)	.08^d^
Educational level, n (%)					<.001^d^
Illiterate	9 (12.0)	28 (42.4)	6 (40.0)	43 (27.6)	
Primary school	23 (30.7)	30 (45.5)	7 (46.7)	60 (38.5)	
Junior or senior high school	37 (49.3)	28 (42.4)	1 (6.7)	46 (29.5)	
College	6 (8.0)	0 (0.0)	1 (6.7)	7 (4.5)	
Smoking, n (%)					.57^d^
No	65 (86.7)	61 (92.4)	14 (93.3)	140 (89.7)	
Yes	7 (9.3)	3 (4.5)	10 (66.7)	10 (6.4)	
Quit	3 (4.0)	2 (3.0)	6 (40.0)	6 (3.8)	
Alcohol consumption, n (%)					.12^d^
No	69 (92.0)	65 (98.5)	15 (100.0)	149 (95.5)	
Yes	6 (8.0)	1 (1.5)	0 (0)	7 (4.5)	
MoCA^e^ score (0-30), median (IQR)	26 (24-27)	19 (17-21)	9 (7-13)	22 (18-25)	<.001^c^

a NCI: no cognitive impairment.

b MCI: mild cognitive impairment.

c Kruskal-Wallis test.

d Fisher exact test.

e MoCA: Montreal Cognitive Assessment.

### Task Performance and Logistic Regression

**Figure 2.**
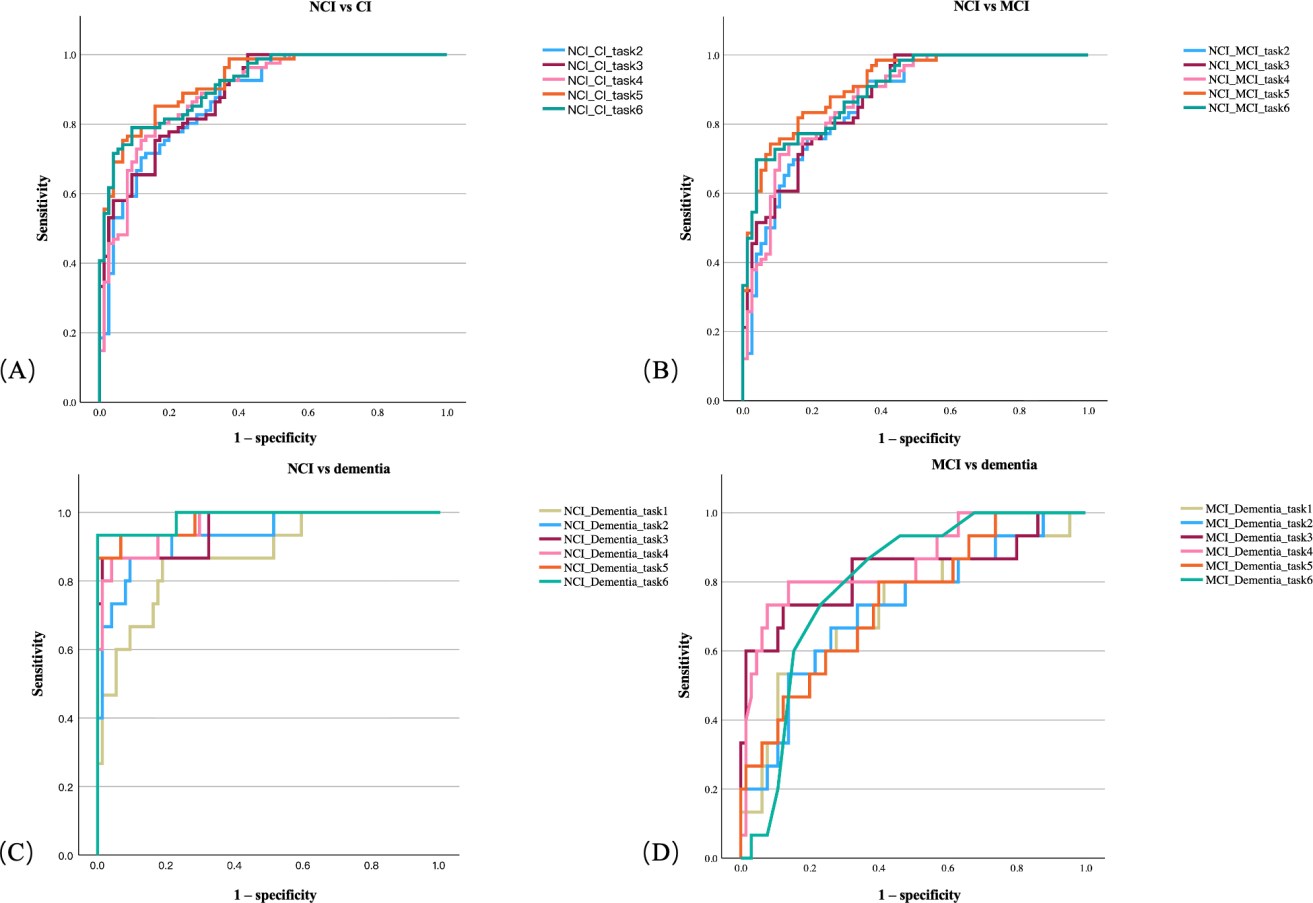
Receiver operating characteristic curve performance of different tasks: (A) different task performance for identification of no cognitive impairment (NCI) vs cognitive impairment (CI), (B) different task performance for identification of NCI vs mild CI (MCI), (C) different task performance for identification of NCI vs dementia, and (D) different task performance for identification of MCI vs dementia.

The results for task performance and logistic regression are shown in Table 2, Multimedia Appendix 1, and Figure 2.

**Table 2.** Logistic regression analysis of task variables for discriminating between cognitive groups (adjusted for educational level and age).

	NCI^a^ vs cognitive impairment			NCI vs MCI^b^			NCI vs dementia			MCI vs dementia		
	Selected variable	β	OR^c^ (95% CI)	Selected variable	β	OR (95% CI)	Selected variable	β	OR (95% CI)	Selected variable	β	OR (95% CI)
Task 1	NS^d^	NS	NS	NS	NS	NS	IES^e^_1^f^	0.940	2.561 (1.271-5.162)	IES_1^g^	0.563	1.757 (1.083-2.848)
Task 2	Go_RT^h^_2^f^	1.420	4.136 (1.876-9.119)	Go_RT_2^f^	1.323	3.755 (1.693-8.330)	Go_RT_2^f^	2.113	8.274 (2.207-31.016)	Go_RT_2^g^	0.570	1.769 (1.122-2.788)
Task 2	FAR^i^_2^g^	0.633	1.884 (1.112-3.192)	FAR_2^g^	0.602	1.825 (1.069-3.118)	—^j^	—	—	—	—	—
Task 3	IES_3^f^	2.159	8.666 (3.244-23.150)	IES_3^f^	2.185	8.891 (3.222-24.534)	IES_3^f^	2.923	16.665 (2.249-123.518)	IES_3^f^	2.278	9.761 (2.313-41.187)
Task 4	GTA^k^_4^f^	1.247	3.480 (1.630-7.433)	GTA_4^f^	1.123	3.074 (1.434-6.590)	GTA_4^f^	2.391	10.920 (1.817-65.629)	Go_RT_4^f^	2.746	15.578 (2.952-82.215)
Task 4	IES_4^f^	2.264	9.618 (3.632-25.469)	IES_4^f^	2.057	7.824 (2.886-21.209)	IES_4^f^	4.349	77.403 (8.050-744.251)	GTA_4^f^	1.721	5.589 (1.287-24.268)
Task 5	IES_5^f^	5.396	220.479 (17.180-2829.514)	IES_5^f^	5.242	189.081 (14.338-2493.478)	IES_5^g^	4.901	134.365 (7.004-2577.765)	Go_RT_5^f^	0.800	2.225 (1.346-3.678)
Task 5	FAR_5^g^	0.608	1.837 (1.010-3.341)	FAR_5^g^	0.691	1.996 (1.074-3.711)	—	—	—	—	—	—
Task 6	Go_RT_6^f^	1.914	6.781 (2.746-16.744)	Go_RT_6^f^	1.820	6.173 (2.483-15.348)	Go_RT_6^g^	1.914	6.781 (2.746-16.744)	FAR_6^f^	0.960	2.613 (1.331-5.131)

a NCI: no cognitive impairment.

b MCI: mild cognitive impairment.

c OR: odds ratio.

d NS: not significant.

e IES: inverse efficiency score.

f *P*<.01.

g *P*<.05.

h RT: reaction time.

i FAR: false alarm rate.

j Not applicable.

k GTA: “go” trial accuracy.

Across all 6 go/no-go tasks, performance on the RT, “go” trial accuracy, inverse efficiency score, and false alarm rate metrics demonstrated a significant declining trend from NCI to dementia (Table 3), with no collinearity issues among metrics (variance inflation factor<10). Compared with participants with NCI, participants with both MCI and dementia exhibited slower RTs, lower accuracy, higher omission rates, and poorer inhibitory control (Table 3).

**Table 3.** Performance on go/no-go tasks across different cognitive groups.

	NCI^a^, median (IQR)	MCI^b^, median (IQR)		Dementia, median (IQR)	*P* value^c^
Task 1					
Go_RT^d^_1 (seconds)	510.28 (480.14-643.33)	515.22 (480.36-795.20)		601.63 (424.22-793.57)	.049
GTA^e^_1 (%)	93.33 (83.33-96.67)	90.00 (82.09-95.00)		86.67 (80.42-94.58)^f^	.03
IES^g^_1	553.86 (512.15-856.74)	606.93 (521.49-1010.24)		729.39 (529.29-1154.87)^f^	.003
FAR^h^_1 (%)	15.00 (10.00-25.00)	20.00 (10.00-30.00)		42.50 (20.00-52.50)^f,i^	.01
Task 2					
Go_RT_2 (seconds)	532.08 (470.87-619.95)	656.22 (562.44-836.66)^f^		705.88 (633.57-1239.25)^f,i^	<.001
GTA_2 (%)	91.67 (85.00-95.00)	85.00 (75.00-94.17)^f^		49.17 (31.25-89.17)^f,i^	<.001
IES_2	600.91 (523.75-761.38)	850.76 (617.57-1355.29)^f^		1751.80 (711.22-3061.99)^f,i^	<.001
FAR_2 (%)	10.00 (5.00-25.00)	20.00 (10.00-45.00)^f^		32.50 (7.50-53.75)^f^	<.001
Task 3					
Go_RT_3 (seconds)	734.56 (701.34-789.56)	880.23 (840.84-922.59)^f^		1026.96 (987.95-1105.29)^f,i^	<.001
GTA_3 (%)	90.00 (85.00-95.00)	71.67 (68.33-77.50)^f^		45.84 (42.92-50.83)^f,i^	<.001
IES_3	838.44 (787.68-910.63)	1318.70 (1208.50-1460.56)^f^		2221.76 (2027.79-2664.91)^f,i^	<.001
FAR_3 (%)	20.00 (7.50-30.00)	50.00 (40.00-62.50)^f^		57.50 (48.75-61.25)^f^	<.001
Task 4					
Go_RT_4 (seconds)	789.45 (745.56-812.73)	930.78 (896.55-975.40)^f^		1026.56 (980.53-1085.51)^f,i^	<.001
GTA_4 (%)	80.00 (72.50-84.17)	68.33 (63.33-71.67)^f^		66.67 (59.58-71.25)^f^	<.001
IES_4	1002.48 (937.43-1135.79)	1490.56 (1389.44-1691.61)^f^		1623.99 (1505.49-1958.68)^f,i^	<.001
FAR_4 (%)	30.00 (15.00-45.00)	55.00 (45.00-65.00)^f^		52.50 (45.00-56.25)^f^	<.001
Task 5					
Go_RT_5 (seconds)	593.53 (513.02-681.63)	705.52 (592.79-879.11)^f^		989.47 (852.59-1576.23)^f,i^	<.001
GTA_5 (%)	93.33 (85.00-96.67)	81.67 (71.67-95.00)^f^		53.33 (23.75-84.59)^f,i^	<.001
IES_5	677.63 (588.29-833.37)	1036.75 (729.19-1205.72)^f^		2244.61 (1116.53-4251.07)^f,i^	<.001
FAR_5 (%)	20.00 (10.00-35.00)	30.00 (10.00-50.00)^f^		42.50 (17.50-70.00)	<.001
Task 6					
Go_RT_6 (seconds)	767.34 (709.16-807.31)	920.67 (885.40-980.56)^f^		1121.00 (1087.95-1158.06)	<.001
GTA_6 (%)	81.67 (76.67-85.84)	68.33 (61.67-73.33)^f^		53.34 (47.92-58.75)^f^	<.001
IES_6	975.92 (912.57-1068.22)	1449.79 (1329.19-1716.65)^f^		2134.88 (2021.61-2437.72)^f^	<.001
FAR_6 (%)	25.00 (17.50-40.00)	45.00 (35.00-55.00)^f^		55.00 (50.00-56.25)^f,i^	<.001

a NCI: no cognitive impairment.

b MCI: mild cognitive impairment.

c Kruskal-Wallis test.

d RT: reaction time.

e GTA: “go” trial accuracy.

f Compared with NCI; *P*<.05.

g IES: inverse efficiency score.

h FAR: false alarm rate.

i Compared with MCI; *P*<.05.

### ROC Results

As shown in Table 2 and Figure 2, we examined the discriminatory ability of different tasks for varying degrees of cognitive decline. The AUC ranges for the diagnostic comparisons were 0.877 to 0.926 for NCI vs cognitive impairment, 0.861 to 0.916 for NCI vs MCI, 0.909 to 0.968 for NCI vs dementia, and 0.724 to 0.855 for MCI vs dementia. Considering accuracy, sensitivity, specificity, and AUC simultaneously, tasks 5 and 6 demonstrated optimal performance in discriminating NCI from cognitive impairment compared to other discrimination models. Task 5 exhibited the most stable performance in discriminating NCI from cognitive impairment, MCI, and dementia (accuracy: 0.837-0.933; sensitivity: 0.742-0.933; specificity: 0.606-0.933; AUC: 0.744-0.977).

In the DeLong test for paired ROC curves, the AUC of task 5 was significantly higher than that of task 2 in discriminating NCI from cognitive impairment (*Z*=–2.129; ΔAUC=–0.048, 95% CI –0.093 to −0.004; *P*=.03) and from MCI (*Z*=–2.182; ΔAUC=–0.055, 95% CI –0.104 to –0.006; *P*=.03). The AUC of task 2 significantly outperformed task 1 when discriminating NCI from dementia (*Z*=–2.05; ΔAUC=–0.049, 95% CI –0.095 to –0.002; *P*=.04).

### Acceptability

In informal interviews, “fun/interesting” emerged as the most frequently mentioned term (76/156, 48.7%), followed by “thinking” (65/156, 41.7%) and “high-tech vibe” (61/156, 39.1%). Participants also expressed perceptions of task difficulty, such as “easy” (57/156, 36.5%), “lagging” (43/156, 27.6%), and “difficult” (13/156, 8.3%). When comparing with standardized scales, participants mentioned the terms “engaging” (43/156, 27.6%) and “novelty” (30/156, 19.2%), with practical application perceptions including “convenient” and “roll out” (Multimedia Appendix 2).

## Discussion

### Principal Findings

#### Overview

This study provides empirical evidence that the increased cognitive load imposed by integrating visual-auditory stimuli and semantic categorization into a go/no-go task effectively amplifies performance differences across cognitive states, thereby supporting its utility as a discriminative tool. This novel approach indirectly assessed the episodic buffer’s ability to form coherent episodic representations, enabling early detection of cognitive decline. Our findings showed that the task complexity incrementally increased from unimodal stimuli (visual or auditory) to multimodal stimuli (audiovisual integration) and from a basic level (concrete objects such as “dog”) to a superordinate level (abstract categories such as “animal”), consistent with our hypothesis. Older adults with cognitive decline exhibited progressively worse performance in these incrementally difficult tasks.

#### Measuring Using Multimodal Stimuli

Unimodal tasks (task 1 and 3), which relied on basic sensory discrimination, did not demonstrate superior overall performance in discriminating between cognitive statuses. Such an observation is likely due to dependence on automatic processing and low-level perception, yielding similar performance across groups. In contrast, both cross-modal matching tasks (tasks 5 and 6) performed well; in particular, they outperformed the auditory-only MoCA-attention cognitive domain (AUC=88.7%, 95% CI 88.0%‐89.4%), suggesting that multisensory integration is a reliable biomarker for cognitive impairment [39]. This is supported by findings from The Irish Longitudinal Study on Ageing, which reported broad associations between multisensory integration and multiple cognitive domains rather than links to any specific subdomain [40]. This implies that measuring multisensory integration could serve as a window into global cognition. Furthermore, as noted previously, multisensory stimulation may serve as a compensatory strategy for older adults, enhancing behavioral performance in those with NCI [26]. However, this compensatory mechanism is limited as behavioral responses are governed by the central executive system, which may account for the more pronounced deficits observed in individuals with progressive cognitive decline. Bayesian integration theory further supports this premise, proposing that perceptual reliability weighting affects audiovisual integration. In other words, the brain optimally combines sensory inputs based on their relative reliability, but cognitive decline may change this weighting process [41]. Apart from that, some scholars regard temporal synchrony and functional connectivity as 2 primary mechanisms that facilitate audiovisual integration [42]. The integration time window posits 2 sequential, interdependent stages: initial parallel peripheral processing of stimuli followed by central processes involving audiovisual integration and motor response initiation [43]. This process may help explain impairments observed in cognitive decline. Previous research indicates that patients with MCI and Alzheimer disease exhibit delayed audiovisual integration compared to older adults with normal cognitive function, with MCI populations perceiving more sound-induced flash illusions [27]. However, patients with MCI and Alzheimer disease experience attentional deficits, and these patients become distracted by irrelevant information, demonstrating impaired attention-switching speed [44]. This aligns with the theory by Baddeley [10]. Impaired performance of the episodic buffer may contribute to working memory deficit, particularly in cognitively declining populations [10]. Therefore, we propose that assessment methods involving multisensory processing can evaluate central executive performance and better identify cognitive decline.

#### Task Complexity

Task 5, rather than the more cognitively demanding task 6, exhibited the most stable discriminative performance across cognitive states. This suggests that excessive complexity may overwhelm the limitations of the episodic buffer and central executive system. A study indicated that older adults tend to adopt conservative action strategies when performing complex tasks, reflecting speed-accuracy trade-offs. Specifically, they adjust their decision boundaries to increase response caution, which results in prolonged RT as a means to enhance or maintain accuracy [45]. However, a study by Chen et al [46] showed different results, with MCI and dementia populations potentially exhibiting random performance without deliberation, resulting in shorter RTs when compared to normal groups. Nonetheless, the adaptive training trials at the beginning of each task in this study could, to some extent, strengthen participants’ understanding of the tasks and avoid performance variability. However, such training for task 6, which placed the highest demands on central executive resources, may still have been insufficient, leading participants to adopt response strategies that influenced the outcomes. According to the Yerkes-Dodson law (an arousal-modulated mechanism) [47], task 5 likely reaches the peak of the inverted U-shaped curve, enhancing arousal and performance discrimination, whereas the increased cognitive load imposed by task 6 exceeds this optimum, resulting in overload and diminished utility. This may also explain why task 5 demonstrated greater stability than task 6. The findings further suggest that task difficulty must be carefully calibrated to avoid overloading the episodic buffer, which could undermine measurement consistency. Additionally, the fixed task order (task 1 to task 6) used in this study means that the decline in performance on task 6 may be partially attributable to time-on-task effects, manifested as decreased attention and less effective decision-making [48]. Previous research has shown that tasks shorter than 15 minutes are unlikely to induce significant fatigue, whereas subjective fatigue increases after 20 minutes of task engagement [49]. Although rest breaks were provided between tasks, this potential confounder should be considered when interpreting the results. Future studies should use task randomization or repeated measurements to rule out this alternative explanation.

### Prospective Clinical Application

At present, the go/no-go task is typically used as an experimental paradigm in conjunction with other objective indicators and has not been widely adopted for screening purposes. In contrast, traditional instruments such as the Mini-Mental State Examination and MoCA require 5 to 15 minutes for administration [24,35]. These traditional tools require trained assessors and additional time and workload and could introduce potential bias from personnel differences [5]. Regarding diagnostic accuracy, our tool demonstrated a performance comparable to that of emerging digital screening instruments, including VR and voice-based tools (AUC=0.83-0.89; sensitivity=0.79-0.83; specificity=0.67-0.91) [7,8,50]. Nevertheless, in comparison with these tools, our novel tool effectively retains the diagnostic benefits of multisensory stimulation while eliminating adverse physical effects and high scene production costs [5-7].

Notably, after accounting for the influence of educational level, the tasks retained their independent predictive value. Within the tasks with independent predictive value, task 5, as the best-performing task, can be completed within 3 minutes. Thus, the use of task 5 as a stand-alone tool for efficient and rapid screening in community settings is highly promising. Task 5 can be integrated into various digital platforms (eg, smartphones or tablets) to quickly flag at-risk individuals for follow-up assessments and can also be deployed as a self-administered tool, thereby enabling timely interventions in resource-limited environments.

Results from the informal interview with community-dwelling older adults showed a positive perception of the modified go/no-go task, with nearly half of the participants describing it as interesting. This favorable experience is important for promoting community-based screening and aligns with previous reports of older adults’ increasing willingness to use digital tools [51]. Additionally, participants’ subjective evaluations of task difficulty were consistent with the study sample proportions of participants with NCI and dementia, indicating that the task’s difficulty gradient was perceptible.

### Limitations

The uneven distribution of participants with dementia, which accounted for 9.6% (15/156) of the total sample, might lead to wide CIs and present a risk of overfitting. This may be related to our community-based data collection. This potential bias calls for future validation studies expanding the sample size of older adults with dementia. Additionally, this study did not repeat measures for test-retest reliability. Therefore, we are currently conducting a follow-up study to fully assess the reliability of the modified go/no-go task. Furthermore, as a cross-sectional study, our findings demonstrate associations but cannot determine the tool’s predictive validity for future dementia onset, which requires longitudinal investigation.

### Conclusions

This study modified and validated a novel go/no-go task incorporating audiovisual stimuli and semantic categorization as an effective tool for early detection of cognitive decline. Positive participant feedback on engagement and novelty supports its feasibility. The applicability and reliability of the modified go/no-go task warrant further investigation in a larger-scale community-based setting.

## Supplementary material

10.2196/91165 Multimedia Appendix 1 Diagnostic performance of cognitive tasks for differentiating between cognitive groups.

10.2196/91165 Multimedia Appendix 2 Task perception word cloud map.

## Abbreviations

AUC: area under the curve
MCI: mild cognitive impairment
MoCA: Montreal Cognitive Assessment
NCI: no cognitive impairment
PFC: prefrontal cortex
ROC: receiver operating characteristic
RT: reaction time
VR: virtual reality
