# Supplementary material for: A Novel Combined Audiovisual-Semantic Digital Tool for Early-Stage Cognitive Decline Detection: Development and Validation Study
Source: JMIR Aging. 2026 May 5;9:e91165. doi: 10.2196/91165 (PMC13143155; doi:10.2196/91165)
Supplement: Multimedia Appendix 1 [file aging-v9-e91165-s001.docx]

Table S1. Diagnostic performance of cognitive tasks for differentiating between cognitive groups.

|  | **NCI^a^ vs cognitive impairment** | | | | **NCI vs MCI^b^** | | | | **NCI vs dementia** | | | | **MCI vs dementia** | | | |
| --- | --- | --- | --- | --- | --- | --- | --- | --- | --- | --- | --- | --- | --- | --- | --- | --- |
|  | **Accuracy** | **Sensitivity** | **Specificity** | **AUC^c^ (95% CI)** | **Accuracy** | **Sensitivity** | **Specificity** | **AUC (95% CI)** | **Accuracy** | **Sensitivity** | **Specificity** | **AUC (95% CI)** | **Accuracy** | **Sensitivity** | **Specificity** | **AUC (95% CI)** |
| Task 1 | NS^d^ | NS | NS | NS | NS | NS | NS | NS | 0.820 | 0.867 | 0.811 | 0.87 (0.776-0.974) | 0.825 | 0.533 | 0.892 | 0.724 (0.571-0.877) |
| Task 2 | 0.788 | 0.704 | 0.88 | 0.877 (0.825-0.930) | 0.780 | 0.910 | 0.667 | 0.865 (0.807-0.923) | 0.9 | 0.867 | 0.907 | 0.934 (0.864-0.972) | 0.728 | 0.666 | 0.742 | 0.734 (0.574-0.874) |
| Task 3 | 0.795 | 0.753 | 0.84 | 0.889 (0.840-0.937) | 0.787 | 0.742 | 0.827 | 0.873 (0.818-0.928) | 0.966 | 0.853 | 0.987 | 0.965 (0.898-0.975) | 0.851 | 0.733 | 0.879 | 0.827 (0.682-0.973) |
| Task 4 | 0.820 | 0.802 | 0.842 | 0.892 (0.843-0.942) | 0.81 | 0.742 | 0.867 | 0.876 (0.820-0.932) | 0.944 | 0.867 | 0.96 | 0.964 (0.919-0.968) | 0.851 | 0.8 | 0.864 | 0.855 (0.737-0.973) |
| Task 5 | 0.846 | 0.852 | 0.925 | 0.926 (0.886-0.963) | 0.837 | 0.742 | 0.92 | 0.914 (0.870-0.958) | 0.933 | 0.933 | 0.933 | 0.977 (0.939-0.983) | 0.84 | 0.8 | 0.606 | 0.744 (0.608-0.881) |
| Task 6 | 0.846 | 0.791 | 0.907 | 0.920 (0.876-0.957) | 0.837 | 0.697 | 0.882 | 0.900 (0.852-0.948) | 0.978 | 0.933 | 0.947 | 0.936 (0.901-0.968) | 0.753 | 0.733 | 0.758 | 0.788 (0.681-0.894) |

^a^NCI: no cognitive impairment.

^b^MCI: mild cognitive impairment.

^c^AUC: area under the curve.

^d^NS: not significant.
