# Supplementary figures and images for: A Novel Combined Audiovisual-Semantic Digital Tool for Early-Stage Cognitive Decline Detection: Development and Validation Study
Source: JMIR Aging. 2026 May 5;9:e91165. doi: 10.2196/91165 (PMC13143155; doi:10.2196/91165)

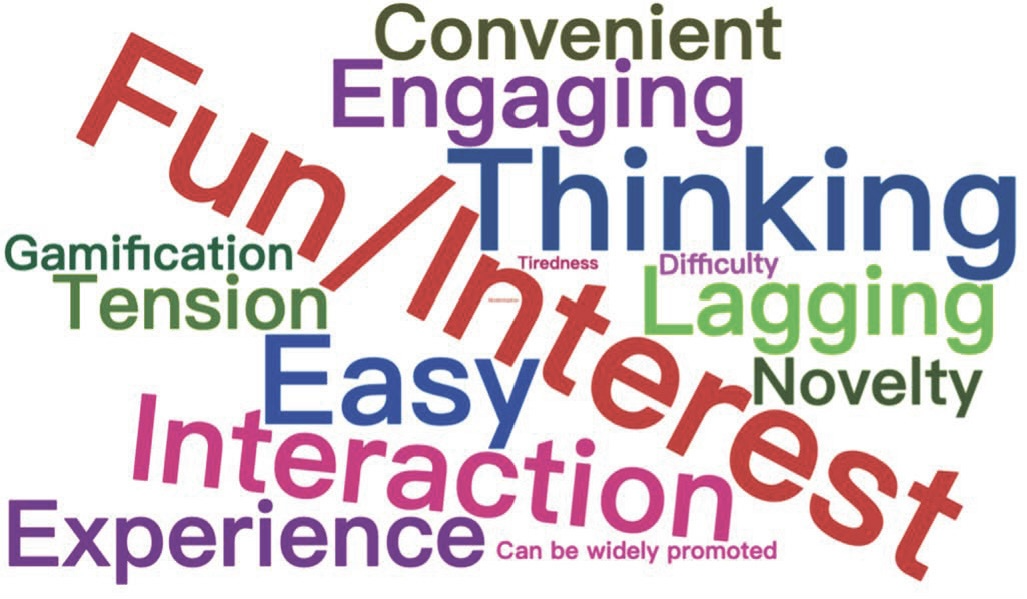

Supplement: Multimedia Appendix 2 [file aging-v9-e91165-s002.png]
